# Screening for odorant receptor genes expressed in *Aedes aegypti* involved in host-seeking, blood-feeding and oviposition behaviors

**DOI:** 10.1186/s13071-022-05196-9

**Published:** 2022-03-04

**Authors:** Meng Ni, Teng Zhao, Hui-xin Lv, Man-jin Li, Dan Xing, Tong-yan Zhao, Chun-xiao Li

**Affiliations:** 1 School of Basic Medical Sciences, Anhui Medical University, Hefei, 230000 China; 2 State Key Laboratory of Pathogen and Biosecurity, Beijing Key Laboratory of Vector Borne and Natural Focus Infectious Disease, Beijing, 100071 China

**Keywords:** *Aedes aegypti*, Olfaction, Odor receptor, Blood-feeding, Oviposition

## Abstract

**Background:**

*Aedes aegypti* is one of the most important vectors of zoonotic diseases worldwide, and its survival and reproductive processes depend heavily on its olfactory system. In this study, the expression levels of all odorant receptor (OR) genes of *Ae. aegypti* were explored during different physiological periods to identify olfactory genes that may be associated with mosquito blood-feeding and the search for oviposition sites.

**Methods:**

Four experimental groups, consisting of *Ae. aegypti* males, pre-blood-feeding females, post-blood-feeding females and post-oviposition females, were established. A total of 114 pairs of primers targeting all messenger RNA encoded by OR genes were designed based on the whole genome of *Ae. aegypti*. The expression of OR genes was evaluated by real-time fluorescence quantitative PCR for relative quantification and the comparison of differences between groups.

**Results:**

A total of 53 differentially expressed OR genes were identified between males and females in *Ae. aegypti* antennae. Also, eight, eight and 13 differentially expressed OR genes were identified in pre- versus post-blood-feeding females, in pre- versus post-oviposition females and in post-blood-feeding versus post-oviposition females, respectively. In addition, 16 OR genes were significantly differentially expressed in multiple physiological periods of the mosquitoes.

**Conclusions:**

A large number of ORs with significant intergroup differences and high expression levels were screened in this study. Some of these genes are reported for the first time, providing possible targets for the development of mosquito control pathways based on the olfactory system.

**Graphical Abstract:**

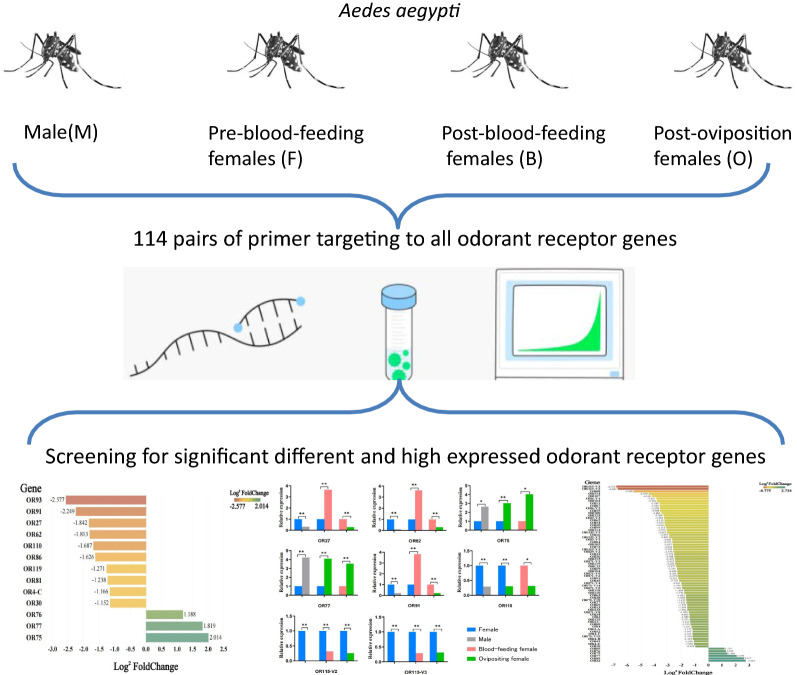

**Supplementary Information:**

The online version contains supplementary material available at 10.1186/s13071-022-05196-9.

## Background

*Aedes aegypti* mosquitoes transmit diseases such as dengue fever, Zika, yellow fever and chikungunya, infecting hundreds of millions of people each year [1]. After mating, female *Ae. aegypti* mainly use smell and temperature to locate the host and suck blood [2]. Mosquitoes mainly rely on their sensitive olfactory system to search for their hosts and for oviposition sites. In recent years, research on the olfactory genes of mosquitoes and the development of new biological control methods have gradually attracted attention [3, 4].

The olfactory system in mosquitoes consists of odorant-binding proteins (OBPs), odorant receptors (ORs) and olfactory neurons (olfactory sensory neurons [OSNs]). The odors emanating from a host are sensed via olfactory receptors, which are found on the mosquito antennae, maxillary palps, and proboscis. When odor molecules bind to ORs on the dendritic membrane of insect OSNs, facilitated by OBPs, the neurons are activated to convert external chemical stimuli into electrical signals [5], which in turn trigger a series of subsequent behaviors. In 1999, Clyne et al. [6] and Vosshall et al. [7] discovered the first OR protein in *Drosophila melanogaster*. ORs are proteins with seven transmembrane domains that are encoded by large gene families. Minor structural variations in OR genes, which have accumulated through evolutionary time, give rise to differences in receptor tuning; for example, the enantiomeric specificity of OR8 in *Culex quinquefasciatus* paralogues relies on eight C-terminal amino acids encoded in the final exon of the gene [8].

Different species of mosquitoes have different numbers of corresponding ORs, with 79 ORs indentified in *Anopheles gambiae *[9], 131 in *Ae. Aegypti *[10], 180 in *Cx. Quinquefasciatus *[11] and 158 in *Aedes Albopictus *[12]. The OR genes have been identified in the genomes of two species of *Aedes*, 19 species of *Anopheles* and one species of *Culex*, and the list of ORs continues to grow [13]. The ORs are divided into two classes: traditional ligand-based ORs (ORx)[14] and co-expressing odorant receptors (odorant receptor coreceptor [Orco]); these classes of genes are highly conserved in different insect species [15, 16]. The high conservation and widespread expression of Orco genes across species suggest that Orcos may be an important component of olfactory function. While the spatiotemporal expression profiles and functions of traditional ORs are highly variable and species specific, the expression of different ORs in different mosquitoes is closely related to specific mosquito olfactory-mediated behaviors (host search and host preference)[17]. The ORs may recognize different types of odors or may be specific to a single odor [18, 19]; for example, female *An. gambiae* mosquitoes specifically express AgOR1, which has a host-seeking function [20].

To date, a number of studies have been carried out on the OR genes of *Ae. aegypti*, and the correlation between certain ORs and blood-feeding olfactory behavior has also been explored. The level of AaOR4 expression in *Ae. aegypti* was found to affect preference of this mosquito for human hosts [21]. The expression of AaOR2 responded to indole, which is the main component of human sweat [22], and that of AaOR8 was reported to be more sensitive to (*R*)-1-octen-3-ol than to (*S*)-1-octen-3-ol [23]. In another study, downregulation of AaOR8 and AaOR49 genes (normally expressed in the stalk) resulted in the mosquitoes showing an increase in stalk-probing behavior and longer blood-feeding times, implying that these ORs are involved in the sucking process [24]. In this study, we performed a systematic analysis of OR gene expression in males, non-blood-feeding females, blood-feeding females and post-oviposition females of *Ae. aegypti*, with the aim to explore the OR genes that may influence host-seeking, blood-feeding and oviposition.

## Methods

### Mosquitoes

The *Ae. aegypti* strain used in this study is a laboratory strain that was originally from Jinghong, Yunnan Province (China) and has been kept in the laboratory for 6 years. The mosquitoes were reared at 26 ± 1 °C, 75 ± 5% relative humidity and a light:dark schedule of 14:10 h. The adult mosquitoes were fed with 8% sugar water for 3–5 days after emergence and then fed a blood meal to breed the next generation.

For the study, we used four groups of *Ae. aegypti*: an male-only group (M); a pre-blood-feeding female group (F); a post-blood-feeding female group (B); and a post-oviposition female group (O). The mosquitoes of groups M and F were adult mosquitoes 3–5 days after emergence; those of group B were the females at 1–2 days after a blood meal; and those of group O were females after oviposition. From each group, 10 pairs of antennae were analyzed. Three biological replicates were performed in each group.

### Extraction and reverse transcription of total RNA

Total RNA was extracted from the mosquitoes of groups M, F, B and O using the RNeasy Mini Kit Reagent (Qiagen, Hilden, Germany) according to the manufacturer’s instructions, and the concentration of total RNA was measured by UV spectrophotometry. The Prime ScriptR RTase Kit (Takara Bio. Inc., Kusatsu, Japan ) was used according to the manufacturer’s instructions. The reaction conditions were as follows: 37 °C for 15 min, 85 °C for 5 s and − 20 °C for storage on standby.

### Primer design and synthesis

The *Ae. aegypti* OR gene sequence (GCF_002204515.2) was downloaded from NCBI (https://www.ncbi.nlm.nih.gov/), and 114 pairs of primers targeting all messenger RNAs (mRNAs) encoded by OR genes were designed using the Primer Premier 5.0 software; these were then synthesized by Tianyi Huiyuan and are listed in Additional file 1: Dataset S1.

### Real-time fluorescence quantitative PCR analysis of OR gene expression

The obtained mosquito antennae complementary DNA (cDNA) was used as a template for real-time fluorescent quantitative PCR (qRT-PCR) using the PerfectStart Green qPCR SuperMix Assay Kit (TransGen Biotech Co., Ltd., Beijing, China). The quantification of each sample was repeated three times, and three replicates were performed for each group. The reaction procedure consisted of: 94 °C for 30 s, followed by 40 cycles of 94 °C for 5 s and 60 °C for 34 s. The real-time PCR amplification and lysis curves were confirmed after the reaction. Primers designed according to the housekeeping gene 40S Ribosomal Protein S6 (*RPS6*) gene of *Ae. aegypti* were used as internal reference primers, The applied primer sequences were RPS6-F (5′-CGTCGTCAGGAACGTATCC-3′) and RPS6-R (5′-TTCTTGGCAGCCTTAGCAG-3′).

### Statistical analysis

The calculation of relative target gene expression levels was performed using the 2^−△△Ct^ method (with rsp6 as an internal control). One-way analysis of variance of the experimental data was performed using IBM SPSS Statistics version 21 software (IBM SPSS, Armonk, NY, USA), and multiple comparison analysis was performed when the overall differences were statistically significant. Dunnett T3 tests were used for multiple comparisons when the variance was not uniform, and post hoc Tukey's HSD tests were used for multiple comparisons when the variance was uniform.* P* < 0.05 was considered to indicate a statistically significant difference. Graphs were created using GraphPad Prism 8.3 (GraphPad Software Inc., San Diego, CA, USA) and Tableau 2021.3 (Tableau Software, Seattle, WA, USA) software.

## Results

### Differentially expressed OR genes between male and female mosquitoes

**Fig. 1:**
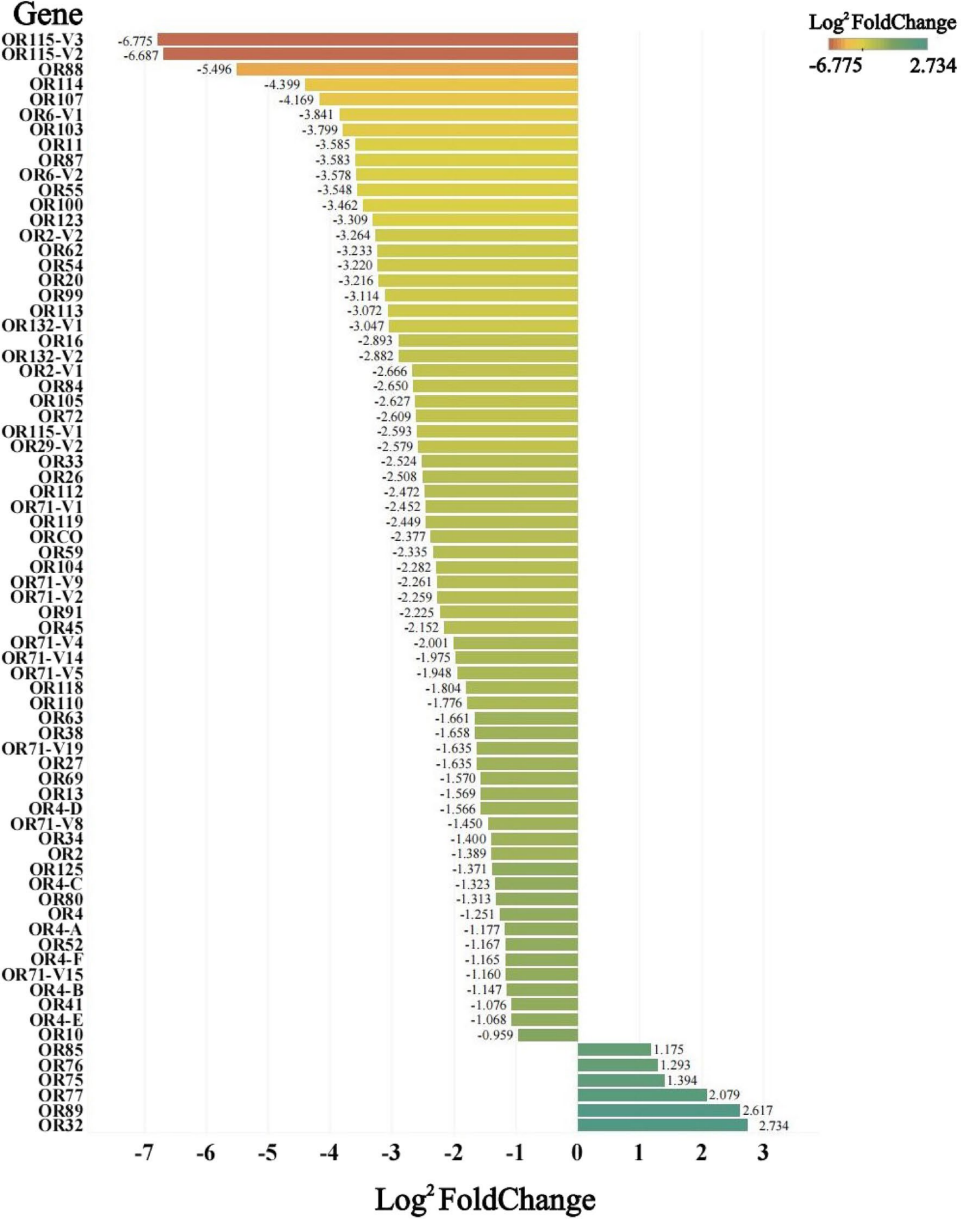
Differences in antennal odorant receptor gene expression between males and females of *Aedes aegypti*

The qRT-PCR results showed that there were 47 *Ae. aegypti* OR genes with lower expression in the antennae of males than females, including OR2, OR10, OR11 and OR13 genes, among others. Among these genes, OR2, OR4, OR6, OR71, OR115 and OR132 genes showed variable splicing. The expression of OR115-V2 and OR115-V3 genes were 103.02- and 109.48-fold higher in female than in male mosquitoes, respectively, and the expression of OR88, OR107 and OR114 genes were 45.14-, 17.99- and 21.10-fold higher in female than in male mosquitoes, respectively. The expression of OR32, OR75, OR76, OR77, OR85 and OR89 genes were higher in male antennae than in female antennae, with expression differences of 6.65-, 2.63-, 2.45-, 4.23-, 2.26- and 6.14 fold, respectively (Fig. 1).

### Differentially expressed OR genes in female mosquitoes before and after blood-feeding

**Fig. 2:**
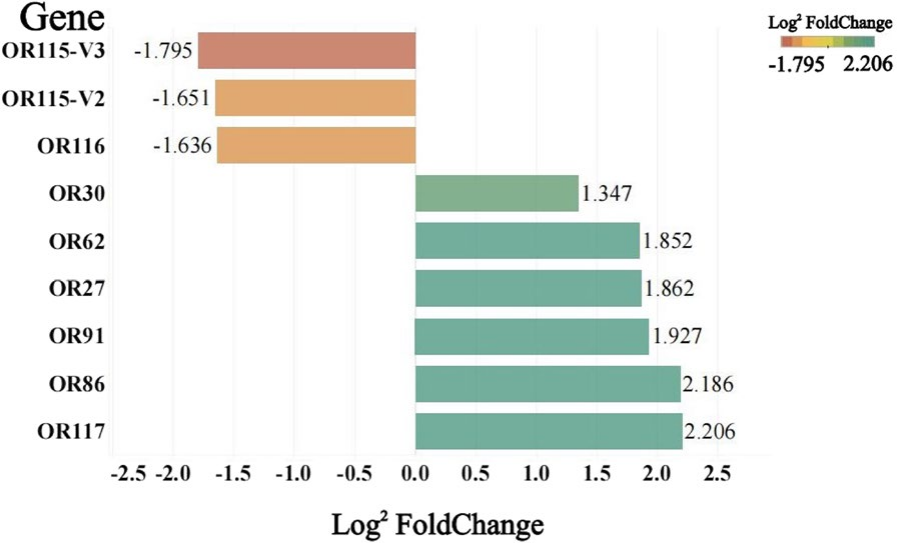
Differences in antennal odorant receptor gene expression between pre-blood-feeding versus post-blood-feeding females of *Ae. aegypti*

The qRT-PCR results showed that there were eight OR genes significantly differentially expressed in *Ae. aegypti* antennae before and after blood-feeding (*P* < 0.05). OR115-V2, OR115-V3 and OR116 genes were downregulated after blood-feeding compared to their levels before blood-feeding, with expression differences of 3.47-, 3.15- and 3.11-fold, respectively. OR27, OR30, OR62, OR86, OR91 and OR117 genes were upregulated after blood-feeding compared with their levels before blood-feeding, with expression differences of 3.64-, 2.54-, 3.61-, 4.55-, 3.80- and 4.61-fold, respectively (Fig. 2).

### Differentially expressed OR genes in female mosquitoes before and after oviposition

**Fig. 3:**
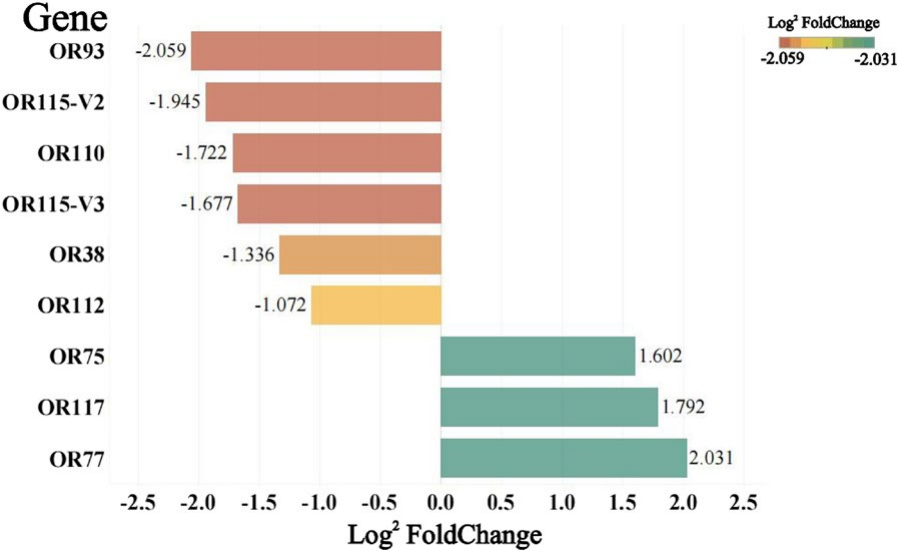
Differences in antennal odorant receptor gene expression between pre-oviposition versus post-oviposition females of *Ae. aegypti*

There were five OR genes that were expressed at lower levels in the antennae of post-oviposition females than in those of pre-oviposition females, including OR38, OR93, OR110, OR112, OR115-V2 and OR115-V3 genes, with expression differences of 2.52-, 4.17-, 3.30-, 2.10-, 3.85- and 3.20-fold, respectively. There were three OR genes that were more highly expressed in the antennae of post-oviposition females than in those of pre-oviposition females, including OR75, OR77 and OR117 genes, with expression differences of 3.04-, 4.09- and 3.46-fold, respectively (Fig. 3).

### Differentially expressed OR genes in female mosquitoes after oviposition versus after blood-feeding

**Fig. 4:**
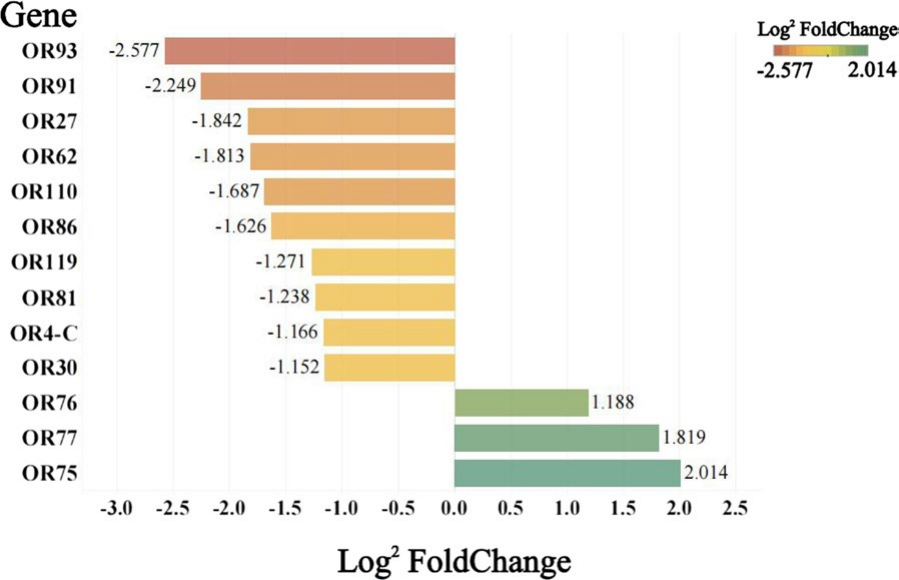
Differences in antennal odorant receptor gene expression between post-blood-feeding females and post-oviposition females of *Ae. aegypti*

There were 10 OR genes that were less highly expressed in the antennae of post-oviposition females than in those of blood-feeding females, including OR4-C, OR27, OR30, OR62, OR81, OR86, OR91, OR93, OR110 and OR119 genes, with expression differences of 2.24-, 3.58-, 2.22-, 3.51-, 2.36-, 3.09-, 4.76-, 5.97-, 3.22- and 2.41-fold, respectively. Three OR genes were more highly expressed in the antennae of post-oviposition females than in those of blood-feeding females, including OR75, OR76 and OR77 genes, with expression differences of 4.04-, 2.28- and 3.53-fold, respectively (Fig. 4).

### OR genes with significantly different expression at different physiological periods of mosquitoes

**Fig. 5:**
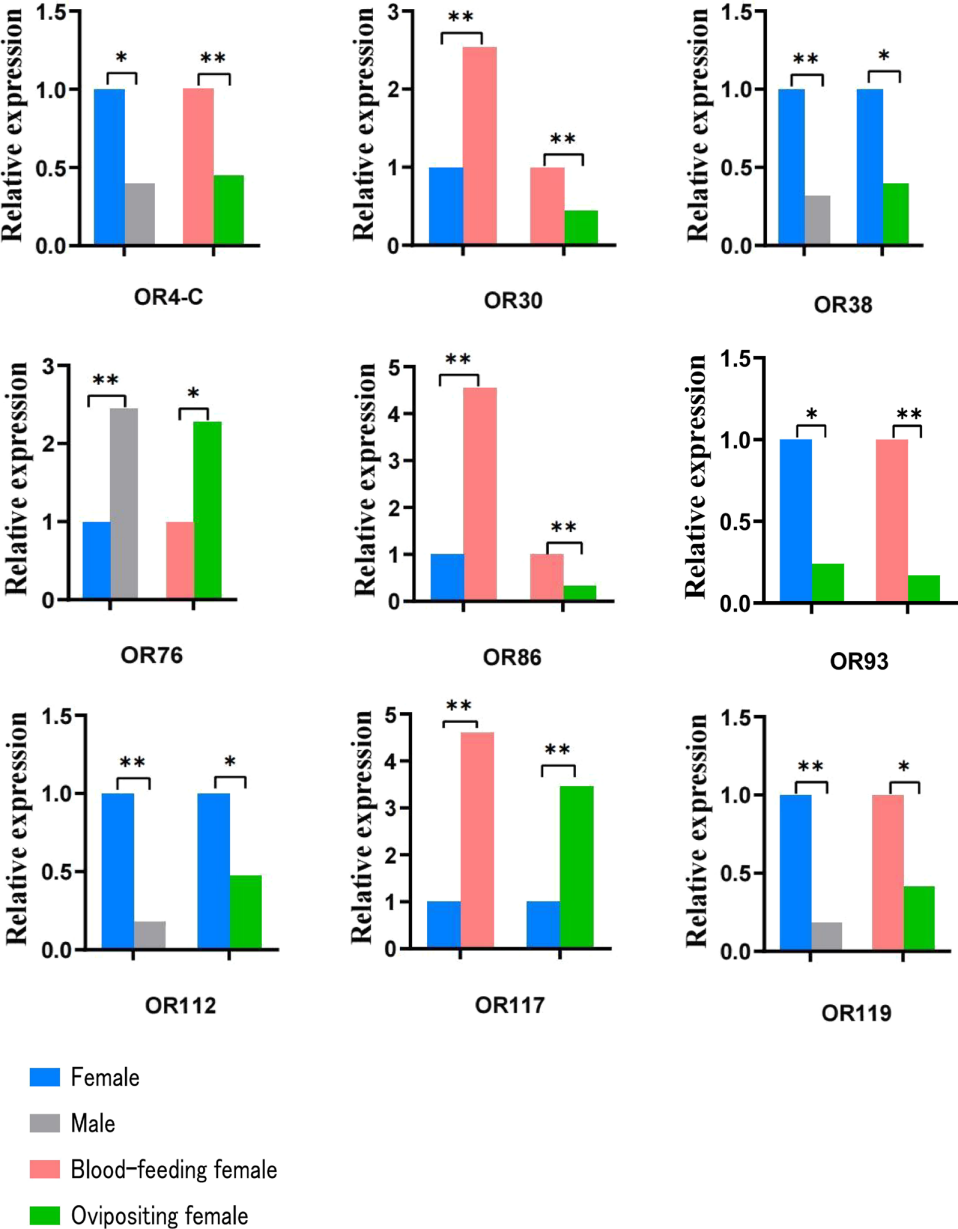
Two of the four groups of comparisons reveal differentially expressed *Ae. aegypti* antennae odorant receptor genes. Asterisks indicate signficant difference at *P < 0.05 and **P < 0.01

Multiple comparison analysis was performed based on the premise that the overall difference was statistically significant (*P* < 0.05). The analysis showed that OR38 and OR112 genes were differentially expressed between male and female *Ae. aegypti* mosquitoes, with 3.16- and 5.55-fold higher expression in females than in males, respectively; the expression of these genes was also different before and after oviposition in females, with 2.52- and 2.10-fold higher expression in pre-oviposition females than in post-oviposition females, respectively. OR93 gene was differentially expressed in female mosquitoes before and after oviposition, with 4.17-fold higher expression in pre-oviposition females than in post-oviposition females; this gene was also differentially in the comparison between the post-oviposition and post-blood-feeding groups of females, with 5.97-fold higher expression in blood-feeding females than in post-oviposition females. OR117 gene was differentially expressed in female mosquitoes before and after blood-feeding, with 4.61-fold higher expression in blood-feeding females than in non-blood-feeding females; this gene was also differentially expressed in females before and after oviposition, with 3.46-fold higher expression in pre-oviposition females than in post-oviposition females. OR4-C, OR76 and OR119 genes were differentially expressed in *Ae. aegypti* mosquitoes between males and females, with 2.50-, 0.41- and 5.46-fold higher expression in females than in males, respectively. Differences in the expression of these genes were also observed in the comparison between the post-oviposition and post-blood feeding groups of females, with 2.24-, 0.44- and 2.41-fold higher expression in post-blood-feeding females than in post-oviposition females, respectively. OR30 and OR86 genes were differentially expressed in the female mosquitoes before and after blood-feeding; their expression in blood-fed female mosquitoes was 2.54- and 4.55-fold higher than that in non-blood-feeding females, respectively. These genes were also differentially expressed between the post-oviposition and post-blood-feeding groups, with expression being 2.22- and 3.09-fold higher in blood-feeding female mosquitoes than in post-oviposition females, respectively (Fig. 5).

**Fig. 6:**
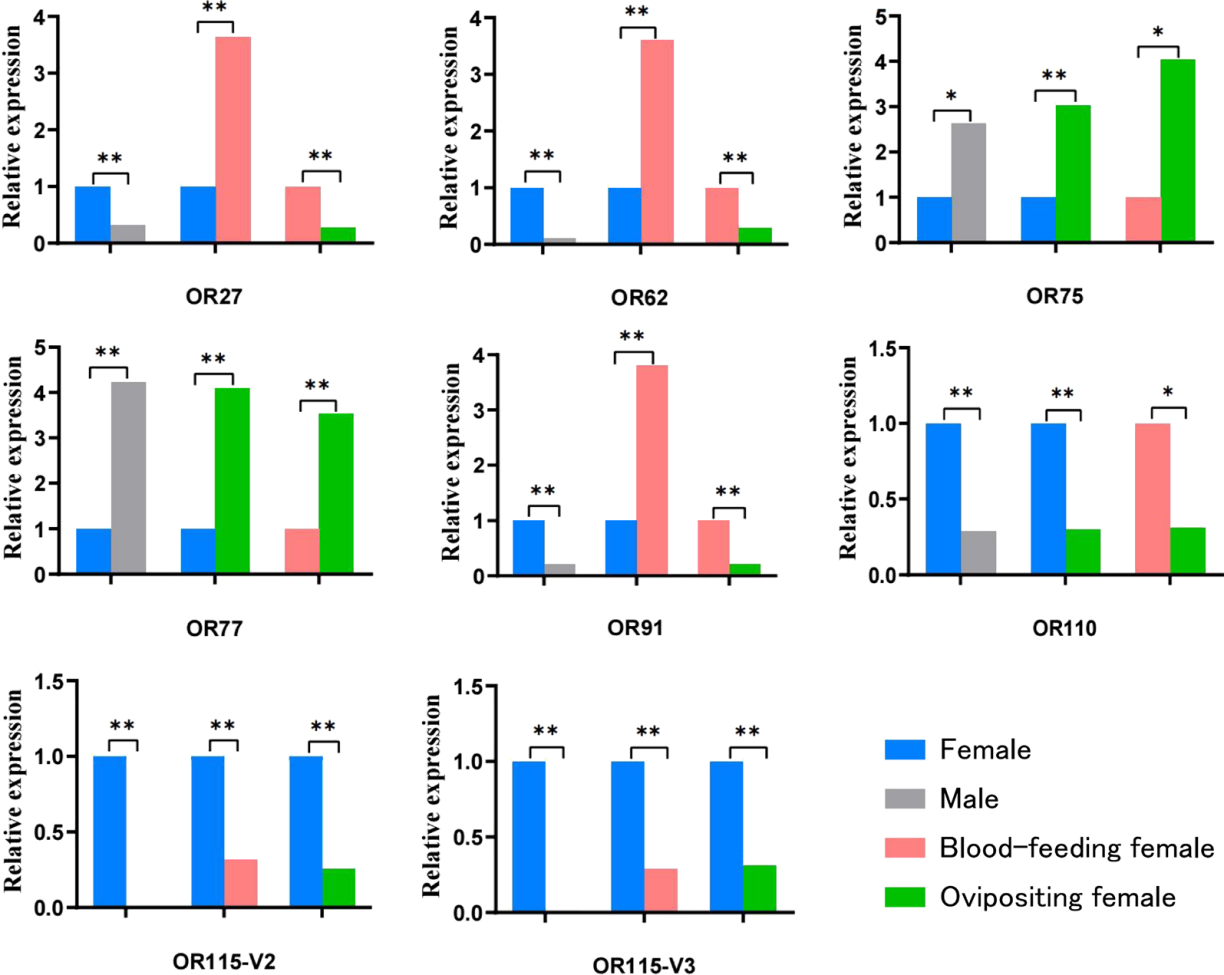
Three of the four groups of comparisons reveal differentially expressed *Ae. aegypti* antennae odorant receptor genes. Asterisks indicate signficant difference at *P < 0.05 and **P < 0.01

OR115-V2 and OR115-V3 genes were differentially expressed not only between males and females of *Ae. aegypti* and between females before and after blood-feeding but also between females before and after oviposition. The expression levels of these genes were 103.02- and 109.48-fold higher in females than in males, 3.47- and 3.15-fold higher in females before versus after blood-feeding and 3.85- and 3.20-fold higher in females before versus after oviposition, respectively. OR75, OR77 and OR110 genes were differentially expressed not only between males and females of *Ae. aegypti* and between females before versus after oviposition, but also in females after oviposition versus after blood-feeding, with 0.38-, 0.24- and 3.42-fold higher expression in females than in males, 0.33-, 0.23- and 3.30-fold higher expression in females before versus after oviposition and 0.25-, 0.28- and 3.22-fold higher expression in females after blood-feeding versus after oviposition, respectively. OR27, OR62 and OR91 genes were differentially expressed not only between males and females of *Ae. aegypti* and before and after blood-feeding in females but also in the comparison between post-oviposition and post-feeding, with 3.11-, 9.40- and 4.68-fold higher expression in females than in males, 3.64-, 3.61- and 3.80-fold higher expression in post-blood-feeding females than in pre-blood-feeding females and 3.58-, 3.51- and 4.76-fold higher expression in blood-feeding females than in post-oviposition females, respectively (Fig. 6).

Detailed relative expression of OR genes in *Ae. aegypti* is shown in Additional file 2: Dataset S2.

## Discussion

*Aedes aegypti* is one of the most important vector organisms in the world, and its survival and reproduction rely heavily on the olfactory system. As one of the key components of the olfactory system, ORs are mainly used to sense chemical information from the environment. In this study, we performed a systematic analysis of the expression of 114 OR genes in male, non-blood-feeding females, blood-feeding females and post-oviposition females of *Ae. aegypti*, and identified a large number of unreported OR genes that may be related to host-searching behavior and oviposition in mosquitoes.

Iatrou et al. [25] compared the expression levels of OR mRNAs in *Anopheles gambiae* antennae and found that the expression of ORs was more easily activated by host odorants in females than in males. In the present study, OR88, OR114, OR115-V2 and OR115-V3 genes in *Ae. aegypti* were found to be highly expressed in female antennae, and their expression levels differed significantly between male and female mosquitoes, suggesting that they may serve as key receptors for host odor detection in females. OR2 and OR4 have been confirmed to be related to the preference of mosquitoes for human hosts [21, 22], we also found differences between male and female mosquitoes in the present study. In a study involving transcriptome sequencing, Matthews et al. [26] found that the expression of OR84, OR87, OR88, OR100, OR103, OR104, OR105, OR112 and OR113 differed between male and female mosquitoes. The results of the present study are consistent with these findings.

Mosquitoes show reduced olfactory activity after blood-feeding and shut down the olfactory response to control energy expenditure to allow midgut blood digestion and ovary development; the reactivation of ORs is delayed after the end of blood digestion to allow females to search for oviposition sites [27]. Based on the finding that OR115-V2, OR115-V3 and OR116 genes were highly expressed before blood-feeding and showed significantly decreased expression after blood-feeding, we hypothesized that these genes are involved in regulating blood-feeding in mosquitoes. Among these genes, downregulated OR116 expression after blood-feeding was also indicated by the sequencing results of Matthews et al. [26].

When mosquitoes have ingested sufficient blood, they need to find suitable oviposition sites to breed their offspring. Gravid mosquitoes use chemosensory cues to select oviposition sites that are suitable for their offspring before depositing their eggs, receiving chemosensory cues via odor receptors mainly on the antennae, but also via contact chemoreceptors on tarsi and mouthparts [28]. The perception of various oviposition attractants increases 24 h after blood-feeding. The upregulation of OR expression may be related to a combination of oviposition lures that induce mosquitoes to find suitable oviposition sites [29]. A comprehensive analysis of the upregulated members of the chemosensory gene family among differentially expressed genes identified before and after a blood meal in *Anopheles sinensis* by Chen et al. [30] revealed that these genes were associated with the precise odor recognition function in this mosquito. The qRT-PCR results of the present study showed that OR27, OR30, OR62, OR86, OR91 and OR117 genes were significantly upregulated after blood-feeding, leading us to presume that these genes are related to the search for suitable oviposition sites based on oviposition attractants by mosquitoes to breed their offspring. Wu et al. [31] found that CquiOR114/117 was associated with blood-feeding in *Cx. quinquefasciatus* by RNA interference and that the mosquito blood-feeding rate was positively correlated with CquiOR114/117 expression. The expression of ORs is regulated in mosquitoes according to their needs in relation to physiological functions.

Chemical cues and signals mediate key behaviors during the different stages in the life-cycle of an adult mosquito. Odor cues originating from mosquito eggs or larvae can potentially be as used as attractants or repellents in vector control strategies. Cuticular hydrocarbons of the mosquito egg have been listed as oviposition pheromones for *Ae. aegypti *[32]. We found that OR38, OR93, OR110, OR112, OR115-V2 and OR115-V3 genes were highly expressed before oviposition and that their expression decreases after oviposition, suggesting that they may play a role in mosquito oviposition in combination with oviposition stimulants. We also found that OR75, OR77 and OR117 genes were upregulated after oviposition, when the mosquito enters the next blood-feeding and oviposition cycle after oviposition. The upregulation of expression may be involved in regulating the next blood-feeding and oviposition cycle of the mosquito.

Our analysis revealed at least two differences among the 16 OR genes, represented by OR4, in the four groups of comparisons (male vs female, pre- vs post-blood-feeding females, pre- vs post-oviposition females and post-blood-feeding females versus post-oviposition females). These results suggest a continuous role of ORs in all physiological periods of the mosquito to enable the use of infochemicals in the environment to locate mates, hosts and oviposition sites throughout the mosquito’s life-cycle.

Highly homologous olfactory proteins in different mosquito species can respond to the same host odor and mosquito oviposition pheromones [33]. AgOR2 is highly conserved among mosquito species [18, 22]. *Culex quinquefasciatus *[34] and *Ae. aegypti *[22] show high OR2 homology, and both species respond to the oviposition attractant indole. Liu et al. [35] simultaneously expressed AalOrco and AalOR10 of *Aedes albopictus* in human embryonic kidney cells and showed that they responded strongly to indole and 1-octen-3-ol, which are major components of human sweat. The ORs CquiOR10 and AsinOR10 recognize 3-methylindole volatilized at oviposition sites [36, 37]. In the present study, the expression of OR2 and OR10 in *Ae. aegypti* females was significantly higher than that in males, with the highest expression found in females after blood-feeding, These two genes are highly homologous to OR2 and OR10 in other mosquito species. It is speculated that *Ae. aegypti* OR2 and OR10 may be involved in *Ae. aegypti* host-seeking, blood-feeding and oviposition.

To summarize, we systematically screened a large number of ORs that may affect the host-seeking, blood-feeding and oviposition behaviors of *Ae. aegypti* mosquitoes, providing a direction forward in the analysis of the structures and interactions of these proteins. The challenge for effective and environmentally friendly mosquito control lies not only in identifying chemicals (hormones) but also in identifying how they bind to mosquito olfactory receptors. Understanding which ORs play a species-specific role in blood-feeding and oviposition behavior could be a key to unraveling the complex interactions of mosquito olfactory receptors in response to chemicals. Only with this knowledge can the formulations be used to enhance the selectivity of odor-based traps and control mosquito-borne disease [38]. ORs with significant intergroup differences and high expression, such as OR93, OR110, OR115, among others, provide possible targets for the development of mosquito control pathways based on the olfactory system.

## Conclusions

In this study, we performed a systematic comparative analysis of the expression of OR genes in *Ae. aegypti* mosquitoes, A large number of ORs with significant intergroup differences and high expression levels were found. OR88, OR107, OR114,OR115-V2 and OR115-V3 genes differed significantly between males and females and may have the function of regulating host-seeking behavior; OR115-V2, OR115-V3 and OR116 genes were found to be downregulated after blood-feeding and may be involved in regulating the blood-feeding behavior; OR27, OR30, OR62, OR86, OR91 and OR117 genes were found to be up regulated after blood-feeding and may be involved in regulating the post-feeding search for oviposition sites; and OR27, OR62, OR91, OR75, OR77 and OR110 may be involved in regulating oviposition behavior. Some of these genes are reported for the first time, providing possible targets for the development of mosquito control based on the olfactory system.

## Supplementary Information

**Additional file 1: Dataset S1**. The PCR primers designed for OR genes in *Ae. aegypti*.

**Additional file 2: Dataset S2**. Relative expression of OR genes in *Ae. aegypti*

## Data Availability

The datasets supporting the conclusions of this article are included within the article.
