# Supplementary material for: Screening for odorant receptor genes expressed in Aedes aegypti involved in host-seeking, blood-feeding and oviposition behaviors
Source: Parasit Vectors. 2022 Mar 4;15:71. doi: 10.1186/s13071-022-05196-9 (PMC8895831; doi:10.1186/s13071-022-05196-9)
Supplement: Supplementary file 1 — Additional file 1: Dataset S1. The PCR primers designed for OR genes in Ae. aegypti. [file 13071_2022_5196_MOESM1_ESM.docx]

Table 1 The 114 PCR primers designed for OR gene in *Aedes aegypti*

| Gene Description | Gene name | Sequences of primers（5′→3′) | |
| --- | --- | --- | --- |
| odorant receptor 2 (Or2) transcript variant1, mRNA | NM_001358323.1 | OR2-V1-F | 5'-ATCAGTGCCTGCTTCGTTAC-3' |
|  |  | OR2-V1-R | 5'-AATCCGAACAGGGCACAGGT-3' |
| odorant receptor 2 (Or2) transcript variant2, mRNA | NM_001358951.1 | OR2-V2-F | 5'-ATCAGTGCCTGCTTCGTTAC-3' |
|  |  | OR2-V2-R | 5'-AATCCGAACAGGGCACAGGT-3' |
| odorant receptor (Or4) mRNA, Or4-A allele | NM_001358193.1 | OR4-F | 5'-GGGAACTCACAGATAAGGAC-3' |
|  |  | OR4-R | 5'-TTGAACGCCGACACCATTAT-3' |
| odorant receptor (Or4) mRNA, Or4-B allele | KF801614.1 | OR4-A-F | 5'-GGGAACTCACAGATAAGGAC-3' |
|  |  | OR4-A-R | 5'-AACGCCGACACCATTATGAT-3' |
| odorant receptor (Or4) mRNA, Or4-F allele | KF801615.1 | OR4-B-F | 5'-GGGAACTCACAGATAAGGAC-3' |
|  |  | OR4-B-R | 5'-GTAACCACCCGACCGTAGAT-3' |
| odorant receptor (Or4) mRNA, Or4-C allele | KF801617.1 | OR4-C-F | 5'-GCTCACAGATAAGGACCGTT-3' |
|  |  | OR4-C-R | 5'-GAAAGTTGAACGCCGACACC-3' |
| odorant receptor (Or4) mRNA, Or4-E allele | KF801618.1 | OR4-D-F | 5'-AGAAAAGGACCGTTCCATAC-3' |
|  |  | OR4-D-R | 5'-AGTCGGATTGAAAGGGAACC-3' |
| odorant receptor (Or4) mRNA, Or4-D allele | KF801619.1 | OR4-E-F | 5'-AGAGAAGGACCGTTACATAC-3' |
|  |  | OR4-E-R | 5'-TTGAAAGGGAACCAAATCAC-3' |
| odorant receptor (Or4) mRNA, Or4-G allele | KF801620.1 | OR4-F-F | 5'-GCTCACAGATAAGGACCGTT-3' |
|  |  | OR4-F-R | 5'-TTGAACGCCGACACCATTAT-3' |
| odorant receptor (Or4) mRNA, Or4-A allele | KF801621.1 | OR4-G-F | 5'-GCTGTTTCCGTATGTGATTT-3' |
|  |  | OR4-G-R | 5'-CGAAGTCAGCAAGCAGAAGA-3' |
| odorant receptor 6 (Or6) transcript variant1, mRNA | NM_001358466.1 | OR6-V1-F | 5'-CATTCTGTCTGCTCACTTAT-3' |
|  |  | OR6-V1-R | 5'-CTTGTTCATCCTATCGTGGC-3' |
| odorant receptor 6 (Or6) transcript variant2, mRNA | NM_001358467.1 | OR6-V2-F | 5'-CATTCTGTCTGCTCACTTAT-3' |
|  |  | OR6-V2-R | 5'-CTTGTTCATCCTATCGTGGC-3' |
| odorant receptor coreceptor (Orco) | NM_001358471.1 | ORCO-F | 5'-CCGTGACCAAGTTCATCTAC-3' |
|  |  | ORCO-R | 5'-GCATCTTAGCCAACGCAATC-3' |
| odorant receptor 10 (Or10) | NM_001358339.1 | OR10-F | 5'-ACCTGCTTCGTAGTCTATCC-3' |
|  |  | OR10-R | 5'-CGGGAAATGTCAAAACAACC-3' |
| odorant receptor 11(Or11) | NM_001358172.1 | OR11-F | 5'-CCACCATTTATCTCATACAG-3' |
|  |  | OR11-R | 5'-CCAGGTTAGCCAACTTGTAG-3' |
| odorant receptor 13 (Or13) | NM_001358180.1 | OR13-F | 5'-TTCGGACAAGGAAGGATTCG-3' |
|  |  | OR13-R | 5'-TCGGGCGAAAAACTCCATCC-3' |
| odorant receptor 15 (Or15) | NM_001358181.1 | OR15-F | 5'-CAGTTTCAAGTGACGATTCT-3' |
|  |  | OR15-R | 5'-TTTCGTATTTGAATCGGTGC-3' |
| odorant receptor 16 (Or16) | NM_001358156.1 | OR16-F | 5'-TTGATGGTGGTCATAAGTTC-3' |
|  |  | OR16-R | 5'-AGGACTTTTCCATTTGCGAC-3' |
| odorant receptor 20 (Or20) | NM_001358155.1 | OR20-F | 5'-CGGAGTTGTATGGTTTGATT-3' |
|  |  | OR20-R | 5'-GCCCAGTGGCATTAAGGAGA-3' |
| odorant receptor 21 (Or21) | NM_001358176.1 | OR21-F | 5'-CTTTTTCTATGTGGTGGTTG-3' |
|  |  | OR21-R | 5'-TGTTATTTGCTGCTCACTGG-3' |
| odorant receptor 22 (Or22) | NM_001358157.1 | OR22-F | 5'-TACGTACTTCCGGTGGGCTA-3' |
|  |  | OR22-R | 5'-AGCCAATGTACACCAAGCCA-3' |
| odorant receptor 24 (Or24) | NM_001358149.1 | OR24-F | 5'-TACGGAAGACCTTATCAACT-3' |
|  |  | OR24-R | 5'-TGACGGGTTCTTGAGCTGAC-3' |
| odorant receptor 25 (Or25) | NM_001358150.1 | OR25-F | 5'-TCATCTGCGGTCTCTATCTT-3' |
|  |  | OR25-R | 5'-CTCCGCTGGTTCTATCAACG-3' |
| odorant receptor 26 (Or26) | NM_001358164.1 | OR26-F | 5'-GGAGACGCTGTATGAGTGTT-3' |
|  |  | OR26-R | 5'-GCCATAAGTTGCCGCTTGAA-3' |
| odorant receptor 27 (Or27) | NM_001358165.1 | OR27-F | 5'-GTTACCTGGTCAAAATCTAC-3' |
|  |  | OR27-R | 5'-GCTGAGAAACTGGACAGATG-3' |
| odorant receptor 28 (Or28) | NM_001358166.1 | OR28-F | 5'-GTTTACAACGACGAGAAGCC-3' |
|  |  | OR28-R | 5'-ACCAGTGGAACCAGGCTCAT-3' |
| odorant receptor 29 (Or29) transcript variant1, mRNA | NM_001358147.1 | OR29-V1-F | 5'-GGTCGGAAACAATATCCTTC-3' |
|  |  | OR29-V1-R | 5'-AAAAGTTGAACCATCCGCAC-3' |
| odorant receptor 29 (Or29) transcript variant2, mRNA | NM_001358954.1 | OR29-V2-F | 5'-GGTCGGAAACAATATCCTTC-3' |
|  |  | OR29-V2-R | 5'-AAAAGTTGAACCATCCGCAC-3' |
| odorant receptor 30 (Or30) | NM_001358183.1 | OR30-F | 5'-TCACATTTTCGTCTACTCCA-3' |
|  |  | OR30-R | 5'-TAGGATGATTCGTTGTTCGC-3' |
| odorant receptor 31 (Or31) | NM_001358160.1 | OR31-F | 5'-TGCGGGCTCAAGTTC-3' |
|  |  | OR31-R | 5'-AGCAACGTACGAGCCAG-3' |
| odorant receptor 32 (Or32) | NM_001358145.1 | OR32-F | 5'-CGGCGATAGTCTACATAGTT-3' |
|  |  | OR32-R | 5'-TGCAACTCCCACAACCACTT-3' |
| odorant receptor 33 (Or33) | NM_001358189.1 | OR33-F | 5'-CGAGCAAAGTATCCAACAGA-3' |
|  |  | OR33-R | 5'-CTTGACCTCCTATCCCGTCG-3' |
| odorant receptor 34 (Or34) | NM_001358623.1 | OR34-F | 5'-CGGTTCAGCAAAGTTATGTT-3' |
|  |  | OR34-R | 5'-CGGGACCCAGAAGAACCAAT-3' |
| odorant receptor 36 (Or36) | NM_001358146.1 | OR36-F | 5'-GTTCCGTGAAGTAAAACTAC-3' |
|  |  | OR36-R | 5'-CGAACGAGGTTTGAAAATAG-3' |
| odorant receptor 38 (Or38) | NM_001358107.1 | OR38-F | 5'-GTTCATCGGAGTTTGTCTAC-3' |
|  |  | OR38-R | 5'-TTTCCTCGGTGGTATCAATC-3' |
| odorant receptor 40 (Or40) | NM_001358154.1 | OR40-F | 5'-CCTGATGTTGCTGATAAATG-3' |
|  |  | OR40-R | 5'-AATCTTGAACAGCCGAATGA-3' |
| odorant receptor 41 (Or41) | NM_001358194.1 | OR41-F | 5'-CAAAGGTTCTCGTCAAATAC-3' |
|  |  | OR41-R | 5'-AAACGGCAATATCACTCCGA-3' |
| odorant receptor 42 (Or42) | NM_001358802.1 | OR42-F | 5'-ATGGAGACCGTAGTTCTTCA-3' |
|  |  | OR42-R | 5'-AACACCGAATGGCAAAATCT-3' |
| odorant receptor 43 (Or43) | NM_001358185.1 | OR43-F | 5'-TTGCGTCATACATAGTAACC-3' |
|  |  | OR43-R | 5'-TGACGGAATCAGCATCTATG-3' |
| odorant receptor 44 (Or44) | NM_001358200.1 | OR44-F | 5'-GGTCGTCTCAGAAATCTTCA-3' |
|  |  | OR44-R | 5'-CCGTCGGTAACGCCAGTAAT-3' |
| odorant receptor 45 (Or45) | NM_001358162.1 | OR45-F | 5'-GGCAAAGTATCTGGAGGAAT-3' |
|  |  | OR45-R | 5'-CCCAAGAACTGCCGTAATGT-3' |
| odorant receptor 47 (Or47) | NM_001358202.1 | OR47-F | 5'-CTGTATCATCGCTCTGCCCT-3' |
|  |  | OR47-R | 5'-TCCCACCACCGATTGAAACA-3' |
| odorant receptor 52 (Or52) | NM_001358170.1 | OR52-F | 5'-TTGCTGGGACTGGTAATGAT-3' |
|  |  | OR52-R | 5'-ACGCAGCAAACGGTACGATT-3' |
| odorant receptor 54 (Or54) | NM_001358168.1 | OR54-F | 5'-CCAGAGTGTTCAAGAGTGTT-3' |
|  |  | OR54-R | 5'-CGGCTGATTTGAGTAGATGC-3' |
| odorant receptor 55 (Or55) | NM_001358184.1 | OR55-F | 5'-CGGTACTGTAACTCCAATGA-3' |
|  |  | OR55-R | 5'-TTCCCAGCAACAGGTATTCA-3' |
| odorant receptor 59 (Or59) | NM_001358199.1 | OR59-F | 5'-GGACCTCGGTTTTCAATACT-3' |
|  |  | OR59-R | 5'-TGAAGCCACCAGCATACCAA-3' |
| odorant receptor 60 (Or60) | NM_001358794.1 | OR60-F | 5'-CGTATGAACATTCCCTTGTA-3' |
|  |  | OR60-R | 5'-ACGATCTTCCTCGCAAAATG-3' |
| odorant receptor 61 (Or61) | NM_001358791.1 | OR61-F | 5'-CGCCAGAATACCTATAACAA-3' |
|  |  | OR61-R | 5'-AGAAAGGCAACAGCAATGAT-3' |
| odorant receptor 62 (Or62) | NM_001358789.1 | OR62-F | 5'-TAATCATCGTCTTGTTCAGG-3' |
|  |  | OR62-R | 5'-CGCCTGATTTTGAACGAACT-3' |
| odorant receptor 63 (Or63) | NM_001358171.1 | OR63-F | 5'-TGTTTGTCTACCAGAAGGTG-3' |
|  |  | OR63-R | 5'-TATCAGCCGCCAAAATACCA-3' |
| odorant receptor 64 (Or64) | NM_001358786.1 | OR64-F | 5'-GCTATTATGGATACGCTTTC-3' |
|  |  | OR64-R | 5'-GGTTATGGTAAGGACGCATT-3' |
| odorant receptor 66 (Or66) | NM_001358173.1 | OR66-F | 5'-GTAGTAAAATGAGGGAACTG-3' |
|  |  | OR66-R | 5'-ACCAGTCAGACATTAGAGCA-3' |
| odorant receptor 67 (Or67) | NM_001358781.1 | OR67-F | 5'-TACGACACCAGTGGGATATG-3' |
|  |  | OR67-R | 5'-TTTCGTCCAGTGATGCGTTG-3' |
| odorant receptor 69 (Or69) | NM_001358174.1 | OR69-F | 5'-CGTGTACTATTCAACAGGTC-3' |
|  |  | OR69-R | 5'-CGAGAATCCCCAGTATGTAA-3' |
| odorant receptor 70 (Or70) | NM_001358175.1 | OR70-F | 5'-AGGGGGAAATAGTGTTCATA-3' |
|  |  | OR70-R | 5'-TTGTTAGCAAATAGCCCCAC-3' |
| odorant receptor 71 (Or71) | NM_001358112.1 | OR71-F | 5'-GAAATCTGTTCATTGCTGTG-3' |
|  |  | OR71-R | 5'-GCTTATCGTGCGTTGCTTGT-3' |
| odorant receptor 71 (OR71)AaegOR71V1 | MG593068.1 | OR71V1-F | 5'-GGAAATCTGTTCACTGCTGT-3' |
|  |  | OR71V1-R | 5'-TCGCAAATCAAAGCGTAGCA-3' |
| odorant receptor 71 (OR71)AaegOR71V2 | MG593069.1 | OR71V2-F | 5'-GTCAAGGACCACTGCTACTC-3' |
|  |  | OR71V2-R | 5'-ACGGTCCGAAGCAGCAGTAG-3' |
| odorant receptor 71 (OR71)AaegOR71V4 | MG593070.1 | OR71V4-F | 5'-CGTGCTAAGTGAAATCTTCC-3' |
|  |  | OR71V4-R | 5'-GGTCCGAAGCAGCAGTAGAT-3' |
| odorant receptor 71 (OR71)AaegOR71V5 | MG593071.1 | OR71V5-F | 5'-GAAATCTGTTCATTGCTGTG-3' |
|  |  | OR71V5-R | 5'-GCTTATCGTGCGTTGCTTGT-3' |
| odorant receptor 71 (OR71)AaegOR71V8 | MG593072.1 | OR71V8-F | 5'-GCTAAGTGAAATCTTCCGAC-3' |
|  |  | OR71V8-R | 5'-CCGAAGCAGCAGTAGATGAA-3' |
| odorant receptor 71 (OR71)AaegOR71V9 | MG593073.1 | OR71V9-F | 5'-GCTAAGTGAAATCTTCCGAC-3' |
|  |  | OR71V9-R | 5'-CCGAAGCAGCAGTAGATGAA-3' |
| odorant receptor 71 (OR71)AaegOR71V14 | MG593074.1 | OR71V14-F | 5'-GCTAAGTGAAATCTTCCGAC-3' |
|  |  | OR71V14-R | 5'-CCGAAGCAGCAGTAGATGAA-3' |
| odorant receptor 71 (OR71)AaegOR71V15 | MG593075.1 | OR71V15-F | 5'-GCTAAGTGAAATCTTCCGAC-3' |
|  |  | OR71V15-R | 5'-CCGAAGCAGCAGTAGATGAA-3' |
| odorant receptor 71 (OR71)AaegOR71V19 | MG593076.1 | OR71V19-F | 5'-GCTAAGTGAAATCTTCCGAC-3' |
|  |  | OR71V19-R | 5'-CCGAAGCAGCAGTAGATGAA-3' |
| odorant receptor 72 (Or72) | NM_001358148.1 | OR72-F | 5'-CAGTTTAGCATTTGGGTTGA-3' |
|  |  | OR72-R | 5'-CCAATCCATCTCGCACCTTC-3' |
| odorant receptor 73 (Or73) | NM_001358130.1 | OR73-F | 5'-TGATGAGCCTCCAGCAGAAC-3' |
|  |  | OR73-R | 5'-GCGAGAACAGCGTGATGGGG-3' |
| odorant receptor 74 (Or74) | NM_001358779.1 | OR74-F | 5'-CAAGACTTCGGTATTCTGTT-3' |
|  |  | OR74-R | 5'-CGGAGTATCTTGTTGGGTGT-3' |
| odorant receptor 75 (Or75) | NM_001358776.1 | OR75-F | 5'-ATTTCTACTTCGGTATGTGG-3' |
|  |  | OR75-R | 5'-TCGCATTGGGTGATGTCTAT-3' |
| odorant receptor 76 (Or76) | NM_001358074.1 | OR76-F | 5'-ATTCACCTGGGTCAAGATAC-3' |
|  |  | OR76-R | 5'-CACCAACCAGGAACCACATT-3' |
| odorant receptor 77 (Or77) | NM_001358187.1 | OR77-F | 5'-AGCGGTGATTTCTCTCAGTA-3' |
|  |  | OR77-R | 5'-CACATCACCATCCCTCCGTT-3' |
| odorant receptor 78 (Or78) | NM_001358188.1 | OR78-F | 5'-ATCAAGGTGTTCCTCATTAC-3' |
|  |  | OR78-R | 5'-GTGTCGTGCTGGAATCATAG-3' |
| odorant receptor 79 (Or79) | NM_001358198.1 | OR79-F | 5'-CCGTACCATCCTCTGCGTTA-3' |
|  |  | OR79-R | 5'-CCTGGGTAACGGAATGCGAA-3' |
| odorant receptor 80 (Or80) | NM_001358169.1 | OR80-F | 5'-ACCATTGCTAATAGTGTCCA-3' |
|  |  | OR80-R | 5'-GGTCAGACTGCCCAAATCAT-3' |
| odorant receptor 81 (Or81) | NM_001358186.1 | OR81-F | 5'-CGGATAAAATGGACACTCTA-3' |
|  |  | OR81-R | 5'-AACGCCAAAAACAGTATCGG-3' |
| odorant receptor 84 (Or84) | NM_001358177.1 | OR84-F | 5'-TCCTCTACTTCTCGCTAAAT-3' |
|  |  | OR84-R | 5'-CCTTTCCACATTGTCTTGAG-3' |
| odorant receptor 85 (Or85) | NM_001358195.1 | OR85-F | 5'-AACTTCGTGAACTCAAGACA-3' |
|  |  | OR85-R | 5'-TCGTGGTTGGTAGGAACTGC-3' |
| odorant receptor 86 (Or86) | NM_001358139.1 | OR86-F | 5'-AATCTTATGATACCCCTTCC-3' |
|  |  | OR86-R | 5'-CGGCAAAATACTTTAGGTGG-3' |
| odorant receptor 87 (Or87) | NM_001358336.1 | OR87-F | 5'-ACCGTTGGATGTGTTACTAT-3' |
|  |  | OR87-R | 5'-GTGTCCCATCACAACTTCCA-3' |
| odorant receptor 88 (Or88) | NM_001358337.1 | OR88-F | 5'-TCTATATTGGAGACCCAGAG-3' |
|  |  | OR88-R | 5'-TGGACCAATCGTAATAGCAG-3' |
| odorant receptor 89 (Or89) | NM_001358774.1 | OR89-F | 5'-TGTGAACTCTCAGCGCCAAT-3' |
|  |  | OR89-R | 5'-CTCGAGTCCGGACATAACGG-3' |
| odorant receptor 90 (Or90) | NM_001358773.1 | OR90-F | 5'-CTGAATCACAGTCAAATCAC-3' |
|  |  | OR90-R | 5'-GCCAACGCATGTAATCCTAA-3' |
| odorant receptor 91 (Or91) | NM_001358420.1 | OR91-F | 5'-GCAGTAGTCATGGTCTTTAT-3' |
|  |  | OR91-R | 5'-TGGCTGCTAAATCGTATTGA-3' |
| odorant receptor 93 (Or93) | NM_001358422.1 | OR93-F | 5'-GCCAGCATACATTTTCTCAT-3' |
|  |  | OR93-R | 5'-GAGTCCAACGCAAATCACAA-3' |
| odorant receptor 94 (Or94) | NM_001358421.1 | OR94-F | 5'-TCTGAAGCATTATTTGGGAG-3' |
|  |  | OR94-R | 5'-ACACTGCCACAGACATTCCA-3' |
| odorant receptor 96 (Or96) | NM_001358472.1 | OR96-F | 5'-ACTTATGTGGCAATCAACTG-3' |
|  |  | OR96-R | 5'-GTCCCAGAAAACAGCCAAAG-3' |
| odorant receptor 99 (Or99) | NM_001358450.1 | OR99-F | 5'-CAGCCGTTGCCTGATTAACG-3' |
|  |  | OR99-R | 5'-CGCTCAACATGATTCGCTGG-3' |
| odorant receptor 100 (Or100) | NM_001358317.1 | OR100-F | 5'-ATTACTTGGAGCAGTTGGAT-3' |
|  |  | OR100-R | 5'-TAAAACCCTCCAATGCTGAA-3' |
| odorant receptor 101 (Or101) | NM_001358163.1 | OR101-F | 5'-AGGATACGGTCGGGAAGACA-3' |
|  |  | OR101-R | 5'-CAGGACTCCAGAAGTTGCGT-3' |
| odorant receptor 102 (Or102) | NM_001358620.1 | OR102-F | 5'-TTCAAACTGCCTCATCAACT-3' |
|  |  | OR102-R | 5'-CTGCTGATAACGATGTGCCA-3' |
| odorant receptor 103 (Or103) | NM_001358179.1 | OR103-F | 5'-CAACTCGTTCGCTATTTTAC-3' |
|  |  | OR103-R | 5'-GGAAATGGCAGCAAGTTTTA-3' |
| odorant receptor 104 (Or104) | NM_001358208.1 | OR104-F | 5'-GGACGATGTAGCCAATAAAC-3' |
|  |  | OR104-R | 5'-GCGGTTTGGTTGAGTTTTCT-3' |
| odorant receptor 105 (Or105) | NM_001358209.1 | OR105-F | 5'-GTGGAGATGATTCTTCTGTC-3' |
|  |  | OR105-R | 5'-AATAAAGGCAACGAGCACAT-3' |
| odorant receptor 106 (Or106) | NM_001358808.1 | OR106-F | 5'-ATGGTGCTGACTTTTCTGAT-3' |
|  |  | OR106-R | 5'-AGCGTGGACATTGAAAGAGG-3' |
| odorant receptor 107 (Or107) | NM_001358211.1 | OR107-F | 5'-ATTGGAGGTATCAGAAGTGG-3' |
|  |  | OR107-R | 5'-GGGCAGGTCATTGTTTTCAG-3' |
| odorant receptor 110 (Or110) | NM_001358214.1 | OR110-F | 5'-CGATAAACTGTCTCAGAGGA-3' |
|  |  | OR110-R | 5'-GCCAAAAACGATTAGCATCA-3' |
| odorant receptor 112 (Or112) | NM_001358913.1 | OR112-F | 5'-TCTGGAGATGACACATACGA-3' |
|  |  | OR112-R | 5'-TTTTCGTAGCAGCACAAGGG-3' |
| odorant receptor 113 (Or113) | NM_001358216.1 | OR113-F | 5'-TAGATGGACTGCTGAACGAT-3' |
|  |  | OR113-R | 5'-ACAACCAGCGATGCTTTTGC-3' |
| odorant receptor 114 (Or114) | NM_001358621.1 | OR114-F | 5'-CGATTACCAGTATCAGGAGT-3' |
|  |  | OR114-R | 5'-TTTCTGTGGTGTCGTTTGGT-3' |
| odorant receptor 115 (Or115) transcript variant1, mRNA | NM_001358376.1 | OR115-V1-F | 5'-AACTCACATTGAAGTGGAAC-3' |
|  |  | OR115-V1-R | 5'-CAAAACCTCCACAACGACTT-3' |
| odorant receptor 115 (Or115) transcript variant2, mRNA | NM_001358378.1 | OR115-V2-F | 5'-CAGACCAATAGAGACGAAAT-3' |
|  |  | OR115-V2-R | 5'-CGGTTGTAGTTTGTCCATCA-3' |
| odorant receptor 115 (Or115) transcript variant3, mRNA | NM_001358379.1 | OR115-V3-F | 5'-CAGACCAATAGAGACGAAAT-3' |
|  |  | OR115-V3-R | 5'-CGGTTGTAGTTTGTCCATCA-3' |
| odorant receptor 116 (Or116) | NM_001358419.1 | OR116-F | 5'-CCCACGGTGTCAGACCAAAGA-3' |
|  |  | OR116-R | 5'-TCCTGGTCTTCAACCAAGCG-3' |
| odorant receptor 117 (Or117) | NM_001358424.1 | OR117-F | 5'-TTGGAGTTATGTTGATTGGG-3' |
|  |  | OR117-R | 5'-TGGGTCAAAAACTCTCGCAG-3' |
| odorant receptor 118 (Or118) | NM_001358528.1 | OR118-F | 5'-TCGCTTCAAACTGATTTCAC-3' |
|  |  | OR118-R | 5'-TAGAGCAGAGCCCCAACAGC-3' |
| odorant receptor 119 (Or119) | NM_001358423.1 | OR119-F | 5'-TCTAATAGGAATGCGAACCA-3' |
|  |  | OR119-R | 5'-CGCTTTCAAATGGCTCCAAT-3' |
| odorant receptor 121 (Or121) | NM_001358473.1 | OR121-F | 5'-TCGGTATGAGAAGTAGCACA-3' |
|  |  | OR121-R | 5'-GCAAGCAAAGCCAATCCAAT-3' |
| odorant receptor 122 (Or122) | NM_001358470.1 | OR122-F | 5'-CAACTGCCTGTATGCTTATC-3' |
|  |  | OR122-R | 5'-TGCTGGTGACGAAGTTTCTC-3' |
| odorant receptor 123 (Or123) | NM_001358474.1 | OR123-F | 5'-GAACTTTATCGTGAACAGAC-3' |
|  |  | OR123-R | 5'-TTTGTAACAGAACGAACCGA-3' |
| odorant receptor 125 (Or125) | NM_001358475.1 | OR125-F | 5'-TCGGCGTACATCAAAATACT-3' |
|  |  | OR125-R | 5'-CCAAGCACCCAACGAATCAT-3' |
| odorant receptor 128 (Or128) | NM_001358807.1 | OR128-F | 5'-AAGGAGGAAATCTTGTGGAC-3' |
|  |  | OR128-R | 5'-GGTGGAAATCGTCGTCACAA-3' |
| odorant receptor 132 (Or132) transcript variant1, mRNA | NM_001358468.1 | OR132-V1-F | 5'-TAGTGGCAAGTAGTTATTCC-3' |
|  |  | OR132-V1-R | 5'-CCAGCGAAAGTAGTGTATCC-3' |
| odorant receptor 132 (Or132) transcript variant2, mRNA | NM_001358469.1 | OR132-V2-F | 5'-TAGTGGCAAGTAGTTATTCC-3' |
|  |  | OR132-V2-R | 5'-CCAGCGAAAGTAGTGTATCC-3' |
| odorant receptor 133 (Or133) | NM_001358338.1 | OR133-F | 5'-GGATACTACTTGTTCTATGG-3' |
|  |  | OR133-R | 5'-GTCAGCACATTTATCACCAG-3' |
