# Supplementary material for: Screening for odorant receptor genes expressed in Aedes aegypti involved in host-seeking, blood-feeding and oviposition behaviors
Source: Parasit Vectors. 2022 Mar 4;15:71. doi: 10.1186/s13071-022-05196-9 (PMC8895831; doi:10.1186/s13071-022-05196-9)
Supplement: Supplementary file 2 — Additional file 2: Dataset S2. Relative expression of OR genes in Ae. aegypti [file 13071_2022_5196_MOESM2_ESM.docx]

Table 2 Relative expression of OR gene in *Aedes aegypti*

| **Gene** | *Equality of Variances* | | | M/F | | B/F | | O/F | | O/B | |
| --- | --- | --- | --- | --- | --- | --- | --- | --- | --- | --- | --- |
|  | ***F*** | ***P*** | | *P* | Relative expression | *P* | Relative expression | *P* | Relative expression | *P* | Relative expression |
| **OR2-V1** | 0.574 | | 0.648 | **0.000** | **0.158** | >0.05 | 1.027 | >0.05 | 0.627 | >0.05 | 0.611 |
| **OR2-V2** | 2.363 | | 0.147 | **0.000** | **0.104** | >0.05 | 1.016 | >0.05 | 0.712 | >0.05 | 0.700 |
| **OR4** | 3.744 | | 0.060 | **0.042** | **0.420** | >0.05 | 0.945 | >0.05 | 0.904 | >0.05 | 0.957 |
| **OR4-A** | 5.068 | | 0.023 | **0.024** | **0.442** | >0.05 | 1.202 | >0.05 | 0.866 | >0.05 | 0.721 |
| **OR4-B** | 5.078 | | 0.029 | **0.007** | **0.452** | >0.05 | 1.080 | >0.05 | 0.687 | >0.05 | 0.636 |
| **OR4-C** | 1.612 | | 0.262 | **0.001** | **0.400** | >0.05 | 1.509 | >0.05 | 0.672 | **0.003** | **0.446** |
| **OR4-D** | 0.835 | | 0.511 | **0.001** | **0.338** | >0.05 | 1.006 | >0.05 | 0.628 | >0.05 | 0.624 |
| **OR4-E** | 3.418 | | 0.073 | **0.002** | **0.477** | >0.05 | 1.094 | >0.05 | 0.837 | >0.05 | 0.765 |
| **OR4-F** | 1.441 | | 0.301 | **0.006** | **0.446** | >0.05 | 1.000 | >0.05 | 0.832 | >0.05 | 0.832 |
| OR4-G | 1.406 | | 0.310 | >0.05 | 0.722 | >0.05 | 1.433 | >0.05 | 1.147 | >0.05 | 0.801 |
| **OR6-V1** | 2.347 | | 0.149 | **0.000** | **0.070** | >0.05 | 0.931 | >0.05 | 0.764 | >0.05 | 0.821 |
| **OR6-V2** | 1.226 | | 0.362 | **0.000** | **0.084** | >0.05 | 0.993 | >0.05 | 0.703 | >0.05 | 0.708 |
| **ORCO** | 6.224 | | 0.017 | **0.020** | **0.192** | >0.05 | 1.669 | >0.05 | 1.781 | >0.05 | 0.615 |
| **OR10** | 2.158 | | 0.171 | **0.008** | **0.514** | >0.05 | 1.092 | >0.05 | 0.975 | >0.05 | 0.893 |
| **OR11** | 2.953 | | 0.098 | **0.000** | **0.083** | >0.05 | 0.851 | >0.05 | 0.689 | >0.05 | 0.809 |
| **OR13** | 1.989 | | 0.194 | **0.002** | **0.337** | >0.05 | 0.968 | >0.05 | 0.679 | >0.05 | 0.702 |
| OR15 | 2.803 | | 0.108 | >0.05 | 0.691 | >0.05 | 1.094 | >0.05 | 0.833 | >0.05 | 0.762 |
| **OR16** | 2.870 | | 0.104 | **0.029** | **0.135** | >0.05 | 0.659 | >0.05 | 0.657 | >0.05 | 0.996 |
| **OR20** | 2.088 | | 0.180 | **0.002** | **0.108** | >0.05 | 0.619 | >0.05 | 0.656 | >0.05 | 1.061 |
| OR21 | 0.531 | | 0.674 | >0.05 | 0.907 | >0.05 | 0.930 | >0.05 | 0.625 | >0.05 | 0.672 |
| OR22 | 1.229 | | 0.361 | >0.05 | 0.676 | >0.05 | 0.778 | >0.05 | 1.040 | >0.05 | 1.337 |
| OR24 | 1.851 | | 0.216 | >0.05 | 0.916 | >0.05 | 1.320 | >0.05 | 0.956 | >0.05 | 0.725 |
| OR25 | 1.022 | | 0.433 | >0.05 | 0.606 | >0.05 | 1.418 | >0.05 | 1.258 | >0.05 | 0.887 |
| **OR26** | 1.009 | | 0.438 | **0.002** | **0.176** | >0.05 | 1.507 | >0.05 | 1.455 | >0.05 | 0.966 |
| **OR27** | 0.319 | | 0.811 | **0.007** | **0.322** | **0.003** | **3.636** | >0.05 | 1.014 | **0.003** | **0.279** |
| OR28 | 3.401 | | 0.074 | >0.05 | 0.977 | >0.05 | 0.683 | >0.05 | 1.091 | >0.05 | 1.598 |
| OR29-V1 | 5.022 | | 0.030 | >0.05 | 0.757 | >0.05 | 1.096 | >0.05 | 1.855 | >0.05 | 1.692 |
| **OR29-V2** | 1.758 | | 0.242 | **0.000** | **0.167** | >0.05 | 0.725 | >0.05 | 0.900 | >0.05 | 1.241 |
| **OR30** | 1.679 | | 0.248 | >0.05 | 0.618 | **0.012** | **2.543** | >0.05 | 1.144 | **0.025** | **0.450** |
| OR31 | 6.228 | | 0.017 | >0.05 | 0.677 | >0.05 | 1.501 | >0.05 | 0.878 | >0.05 | 0.625 |
| **OR32** | 4.229 | | 0.053 | **0.007** | **6.651** | >0.05 | 1.509 | >0.05 | 1.338 | >0.05 | 0.887 |
| **OR33** | 1.776 | | 0.229 | **0.004** | **0.174** | >0.05 | 0.992 | >0.05 | 1.123 | >0.05 | 1.132 |
| **OR34** | 0.588 | | 0.640 | **0.015** | **0.379** | >0.05 | 0.628 | >0.05 | 0.661 | >0.05 | 1.054 |
| OR36 | 3.623 | | 0.084 | >0.05 | 1.313 | >0.05 | 1.162 | >0.05 | 1.219 | >0.05 | 1.049 |
| **OR38** | 0.767 | | 0.544 | **0.001** | **0.317** | >0.05 | 0.631 | **0.005** | **0.396** | >0.05 | 0.628 |
| OR40 | 6.705 | | 0.014 | >0.05 | 0.699 | >0.05 | 0.946 | >0.05 | 0.677 | >0.05 | 0.716 |
| **OR41** | 2.757 | | 0.112 | **0.039** | **0.474** | >0.05 | 1.522 | >0.05 | 1.482 | >0.05 | 0.974 |
| OR42 | 2.178 | | 0.169 | >0.05 | 1.397 | >0.05 | 0.648 | >0.05 | 0.796 | >0.05 | 1.229 |
| OR43 | 1.032 | | 0.429 | >0.05 | 0.809 | >0.05 | 0.945 | >0.05 | 1.409 | >0.05 | 1.490 |
| OR44 | 2.955 | | 0.098 | >0.05 | 0.626 | >0.05 | 0.916 | >0.05 | 0.707 | >0.05 | 0.772 |
| **OR45** | 3.947 | | 0.053 | **0.013** | **0.225** | >0.05 | 1.449 | >0.05 | 0.948 | >0.05 | 0.654 |
| OR47 | 4.082 | | 0.067 | >0.05 | 0.635 | >0.05 | 0.765 | >0.05 | 0.768 | >0.05 | 1.005 |
| **OR52** | 0.583 | | 0.643 | **0.009** | **0.445** | >0.05 | 1.041 | >0.05 | 1.354 | >0.05 | 1.301 |
| **OR54** | 2.549 | | 0.129 | **0.001** | **0.107** | >0.05 | 1.232 | >0.05 | 0.865 | >0.05 | 0.702 |
| **OR55** | 3.644 | | 0.064 | **0.001** | **0.085** | >0.05 | 0.861 | >0.05 | 0.634 | >0.05 | 0.736 |
| **OR59** | 0.361 | | 0.783 | **0.009** | **0.198** | >0.05 | 0.937 | >0.05 | 0.838 | >0.05 | 0.895 |
| OR60 | 1.066 | | 0.416 | >0.05 | 1.756 | >0.05 | 1.055 | >0.05 | 1.020 | >0.05 | 0.967 |
| OR61 | 1.614 | | 0.261 | >0.05 | 0.936 | >0.05 | 0.997 | >0.05 | 1.067 | >0.05 | 1.070 |
| **OR62** | 2.207 | | 0.165 | **0.000** | **0.106** | **0.007** | **3.609** | >0.05 | 1.027 | **0.008** | **0.285** |
| **OR63** | 2.447 | | 0.139 | **0.000** | **0.316** | >0.05 | 1.167 | >0.05 | 1.623 | >0.05 | 1.390 |
| OR64 | 1.190 | | 0.373 | >0.05 | 0.733 | >0.05 | 1.125 | >0.05 | 0.847 | >0.05 | 0.753 |
| OR66 | 2.819 | | 0.107 | >0.05 | 0.686 | >0.05 | 1.412 | >0.05 | 1.377 | >0.05 | 0.976 |
| OR67 | 1.033 | | 0.428 | >0.05 | 1.278 | >0.05 | 1.174 | >0.05 | 0.823 | >0.05 | 0.701 |
| **OR69** | 0.203 | | 0.892 | **0.005** | **0.337** | >0.05 | 0.695 | >0.05 | 0.753 | >0.05 | 1.083 |
| OR70 | 0.889 | | 0.487 | >0.05 | 0.830 | >0.05 | 0.793 | >0.05 | 0.677 | >0.05 | 0.759 |
| OR71 | 1.762 | | 0.232 | >0.05 | 0.918 | >0.05 | 0.860 | >0.05 | 0.680 | >0.05 | 0.791 |
| **OR71-V1** | 3.749 | | 0.060 | **0.000** | **0.183** | >0.05 | 0.727 | >0.05 | 0.610 | >0.05 | 0.840 |
| **OR71-V2** | 0.117 | | 0.948 | **0.002** | **0.209** | >0.05 | 0.746 | >0.05 | 0.643 | >0.05 | 0.862 |
| **OR71-V4** | 2.964 | | 0.097 | **0.001** | **0.250** | >0.05 | 0.807 | >0.05 | 0.658 | >0.05 | 0.815 |
| **OR71-V5** | 0.266 | | 0.848 | **0.001** | **0.259** | >0.05 | 0.752 | >0.05 | 0.695 | >0.05 | 0.925 |
| **OR71-V8** | 1.491 | | 0.289 | **0.002** | **0.366** | >0.05 | 0.811 | >0.05 | 0.681 | >0.05 | 0.839 |
| **OR71-V9** | 1.964 | | 0.198 | **0.001** | **0.209** | >0.05 | 0.732 | >0.05 | 0.62 | >0.05 | 0.847 |
| **OR71-V14** | 4.352 | | 0.043 | **0.000** | **0.254** | >0.05 | 0.827 | >0.05 | 0.649 | >0.05 | 0.785 |
| **OR71-V15** | 2.742 | | 0.113 | **0.023** | **0.447** | >0.05 | 0.746 | >0.05 | 0.649 | >0.05 | 0.871 |
| **OR71-V19** | 1.833 | | 0.219 | **0.005** | **0.322** | >0.05 | 0.681 | >0.05 | 0.702 | >0.05 | 1.031 |
| **OR72** | 1.484 | | 0.291 | **0.038** | **0.164** | >0.05 | 1.272 | >0.05 | 1.197 | >0.05 | 0.941 |
| OR73 | 1.573 | | 0.270 | >0.05 | 0.903 | >0.05 | 1.028 | >0.05 | 1.188 | >0.05 | 1.156 |
| OR74 | 2.209 | | 0.165 | >0.05 | 1.271 | >0.05 | 1.554 | >0.05 | 1.237 | >0.05 | 0.796 |
| **OR75** | 2.845 | | 0.105 | **0.021** | **2.628** | >0.05 | 0.751 | **0.011** | **3.035** | **0.003** | **4.039** |
| **OR76** | 0.347 | | 0.793 | **0.022** | **2.450** | >0.05 | 0.670 | >0.05 | 1.527 | **0.031** | **2.279** |
| **OR77** | 2.512 | | 0.132 | **0.018** | **4.226** | >0.05 | 1.159 | **0.020** | **4.088** | **0.032** | **3.527** |
| OR78 | 1.894 | | 0.209 | >0.05 | 0.925 | >0.05 | 1.896 | >0.05 | 1.813 | >0.05 | 0.956 |
| OR79 | 1.947 | | 0.201 | >0.05 | 0.617 | >0.05 | 0.851 | >0.05 | 1.064 | >0.05 | 1.250 |
| **OR80** | 1.438 | | 0.302 | **0.001** | **0.403** | >0.05 | 0.902 | >0.05 | 0.687 | >0.05 | 0.762 |
| **OR81** | 5.583 | | 0.023 | >0.05 | 0.780 | >0.05 | 1.418 | >0.05 | 0.601 | **0.041** | **0.424** |
| **OR84** | 0.450 | | 0.724 | **0.001** | **0.159** | >0.05 | 0.959 | >0.05 | 0.681 | >0.05 | 0.710 |
| **OR85** | 7.527 | | 0.010 | **0.002** | **2.258** | >0.05 | 1.166 | >0.05 | 1.759 | >0.05 | 1.509 |
| **OR86** | 2.675 | | 0.118 | >0.05 | 0.963 | **0.003** | **4.552** | >0.05 | 1.474 | **0.014** | **0.324** |
| **OR87** | 1.766 | | 0.231 | **0.000** | **0.083** | >0.05 | 1.107 | >0.05 | 1.070 | >0.05 | 0.967 |
| **OR88** | 0.867 | | 0.497 | **0.000** | **0.022** | >0.05 | 1.506 | >0.05 | 1.292 | >0.05 | 0.858 |
| **OR89** | 1.744 | | 0.235 | **0.014** | **6.136** | >0.05 | 1.109 | >0.05 | 0.939 | >0.05 | 0.847 |
| OR90 | 7.506 | | 0.010 | >0.05 | 1.432 | >0.05 | 1.011 | >0.05 | 0.746 | >0.05 | 0.737 |
| **OR91** | 0.067 | | 0.976 | **0.002** | **0.214** | **0.005** | **3.803** | >0.05 | 0.800 | **0.002** | **0.210** |
| **OR93** | 0.491 | | 0.698 | >0.05 | 1.715 | >0.05 | 1.432 | **0.013** | **0.240** | **0.004** | **0.168** |
| OR94 | 1.201 | | 0.370 | >0.05 | 0.643 | >0.05 | 0.840 | >0.05 | 0.847 | >0.05 | 1.254 |
| OR96 | 1.402 | | 0.311 | >0.05 | 0.704 | >0.05 | 0.819 | >0.05 | 0.826 | >0.05 | 1.009 |
| **OR99** | 1.152 | | 0.386 | **0.001** | **0.116** | >0.05 | 0.777 | >0.05 | 0.602 | >0.05 | 0.775 |
| **OR100** | 0.955 | | 0.459 | **0.000** | **0.091** | >0.05 | 1.009 | >0.05 | 1.895 | >0.05 | 1.877 |
| OR101 | 0.358 | | 0.785 | >0.05 | 1.563 | >0.05 | 1.654 | >0.05 | 1.204 | >0.05 | 0.728 |
| OR102 | 3.325 | | 0.077 | >0.05 | 1.218 | >0.05 | 0.933 | >0.05 | 0.709 | >0.05 | 0.760 |
| **OR103** | 1.056 | | 0.420 | **0.000** | **0.072** | >0.05 | 1.250 | >0.05 | 1.858 | >0.05 | 1.487 |
| **OR104** | 2.443 | | 0.139 | **0.000** | **0.206** | >0.05 | 1.172 | >0.05 | 1.153 | >0.05 | 0.984 |
| **OR105** | 1.707 | | 0.242 | **0.003** | **0.162** | >0.05 | 1.288 | >0.05 | 0.834 | >0.05 | 0.647 |
| OR106 | 0.141 | | 0.932 | >0.05 | 0.630 | >0.05 | 0.784 | <0.05 | 0.615 | >0.05 | 0.784 |
| **OR107** | 4.660 | | 0.043 | **0.001** | **0.056** | >0.05 | 1.420 | >0.05 | 1.052 | >0.05 | 0.741 |
| **OR110** | 2.301 | | 0.154 | **0.015** | **0.292** | >0.05 | 0.976 | **0.011** | **0.303** | **0.013** | **0.311** |
| **OR112** | 1.547 | | 0.276 | **0.000** | **0.180** | >0.05 | 0.765 | **0.020** | **0.476** | >0.05 | 0.622 |
| OR113 | 4.161 | | 0.047 | >0.05 | 0.822 | >0.05 | 0.816 | >0.05 | 0.693 | >0.05 | 0.848 |
| **OR114** | 2.241 | | 0.161 | **0.000** | **0.047** | >0.05 | 0.652 | >0.05 | 0.729 | >0.05 | 1.118 |
| **OR115-V1** | 4.724 | | 0.035 | **0.001** | **0.166** | >0.05 | 0.620 | >0.05 | 0.626 | >0.05 | 1.009 |
| **OR115-V2** | 5.545 | | 0.024 | **0.000** | **0.010** | **0.006** | **0.318** | **0.002** | **0.260** | >0.05 | 0.816 |
| **OR115-V3** | 2.034 | | 0.198 | **0.000** | **0.009** | **0.001** | **0.288** | **0.002** | **0.313** | >0.05 | 1.086 |
| **OR116** | 2.717 | | 0.115 | >0.05 | 1.587 | **0.002** | **0.322** | >0.05 | 0.604 | >0.05 | 1.876 |
| **OR117** | 0.814 | | 0.521 | >0.05 | 1.171 | **0.000** | **4.612** | **0.001** | **3.463** | >0.05 | 0.751 |
| **OR118** | 0.718 | | 0.569 | **0.019** | **0.286** | >0.05 | 1.179 | >0.05 | 0.892 | >0.05 | 0.756 |
| **OR119** | 0.162 | | 0.919 | **0.000** | **0.183** | >0.05 | 1.583 | >0.05 | 0.656 | **0.005** | **0.414** |
| OR121 | 1.254 | | 0.353 | >0.05 | 1.177 | >0.05 | 0.666 | >0.05 | 1.659 | >0.05 | 1.659 |
| OR122 | 1.359 | | 0.323 | >0.05 | 0.818 | >0.05 | 1.193 | >0.05 | 1.006 | >0.05 | 0.843 |
| **OR123** | 5.034 | | 0.030 | **0.000** | **0.101** | >0.05 | 1.205 | >0.05 | 0.969 | >0.05 | 1.096 |
| **OR125** | 1.186 | | 0.375 | **0.000** | **0.387** | >0.05 | 0.903 | >0.05 | 0.947 | >0.05 | 1.048 |
| OR128 | 0.116 | | 0.948 | >0.05 | 1.411 | >0.05 | 0.612 | >0.05 | 0.707 | >0.05 | 1.156 |
| **OR132-V1** | 0.165 | | 0.917 | **0.000** | **0.121** | >0.05 | 0.701 | >0.05 | 0.808 | >0.05 | 1.152 |
| **OR132-V2** | 0.916 | | 0.475 | **0.000** | **0.136** | >0.05 | 0.704 | >0.05 | 0.860 | >0.05 | 1.222 |
| OR133 | 3.995 | | 0.052 | >0.05 | 1.112 | >0.05 | 1.560 | >0.05 | 1.735 | >0.05 | 1.112 |

P < 0.05 is marked with red color. Males (M), pre-blood--feeding females (F), post-blood-feeding females (B) and post-oviposition females (O). Equality of Variances was tested with Levene’s Test. One-way ANOVA of the experimental data was performed, and multiple comparison analysis was performed when the overall differences were statistically significant. Dunnett T3 tests were used for multiple comparisons when the variance was not uniform, and post hoc Tukey's HSD tests were used for multiple comparisons when the variance was uniform.
